# Circulating Extracellular Vesicles Are Increased in Newly Diagnosed Celiac Disease Patients

**DOI:** 10.3390/nu15010071

**Published:** 2022-12-23

**Authors:** Konstantinos Efthymakis, Giuseppina Bologna, Pasquale Simeone, Laura Pierdomenico, Giulia Catitti, Simone Vespa, Angelo Milano, Domenico De Bellis, Francesco Laterza, Assunta Pandolfi, Caterina Pipino, Michele Sallese, Marco Marchisio, Sebastiano Miscia, Matteo Neri, Paola Lanuti

**Affiliations:** 1 Department of Medicine and Aging Sciences, University “G. d’Annunzio”, Chieti-Pescara, 66100 Chieti, Italy; 2 Digestive Endoscopy and Gastroenterology Unit, SS Annunziata Hospital, ASL2 Abruzzo, 66100 Chieti, Italy; 3 Center for Advanced Studies and Technology (C.A.S.T.), University “G. d’Annunzio”, Chieti-Pescara, 66100 Chieti, Italy; 4 Department of Medical, Oral and Biotechnological Sciences, University “G. d’Annunzio”, Chieti-Pescara, 66100 Chieti, Italy; 5 Department of Innovative Technologies in Medicine & Dentistry, University “G. d’Annunzio” Chieti-Pescara, 66100 Chieti, Italy

**Keywords:** extracellular vesicles, flow cytometry, celiac disease, gluten-free diet regimen (GFD)

## Abstract

Extracellular vesicles (EVs) are a class of circulating entities that are involved in intercellular crosstalk mechanisms, participating in homeostasis maintenance, and diseases. Celiac disease is a gluten-triggered immune-mediated disorder, characterized by the inflammatory insult of the enteric mucosa following local lymphocytic infiltration, resulting in villous atrophy. The goal of this research was the assessment and characterization of circulating EVs in celiac disease patients, as well as in patients already on an adequate gluten-free regimen (GFD). For this purpose, a novel and validated technique based on polychromatic flow cytometry that allowed the identification and enumeration of different EV sub-phenotypes was applied. The analysis evidenced that the total, annexin V+, leukocyte (CD45+), and platelet (CD41a+) EV counts were significantly higher in both newly diagnosed celiac disease patients and patients under GFD compared with the healthy controls. Endothelial-derived (CD31+) and epithelial-derived (EpCAM+) EV counts were significantly lower in subjects under gluten exclusion than in celiac disease patients, although EpCAM+ EVs maintained higher counts than healthy subjects. The numbers of EpCAM+ EVs were a statistically significant predictor of intraepithelial leukocytes (IEL). These data demonstrate that EVs could represent novel and potentially powerful disease-specific biomarkers in the context of celiac disease.

## 1. Introduction

Extracellular vesicles (EVs) are a relatively recently discovered class of circulating bodies, initially thought to represent cellular debris [1,2]. However, different studies conducted on health and disease models showed that they may be involved in intercellular crosstalk mechanisms, participating in homeostasis, and promoting disease onset and progression [3,4]. They have been traditionally classified, according to their size, origin, and functional characteristics, into three major classes: exosomes (50–100 nm), microvesicles (100 nm–2 μm), and apoptotic bodies (1–5 μm) [5]. Exosomes are produced by exocytosis, microvesicles stem from their parental cells by budding and shedding, while apoptotic bodies are released by blebbing during cellular death processes. All these EV sub-types convey specific cargoes consisting of proteins, lipids, genomic DNA, RNAs, and miRNAs [6,7]. Given that these EV classes are overlapping in size, their classification, which is mainly based on dimension, does not fit EV heterogeneity [7,8]. Thus, the International Society of Extracellular Vesicles (ISEV) endorsed the use of the term “extracellular vesicles’’ for all EV types and proposed a generic classification for “small EVs” (100–200 nm) and “medium/large EVs” (>200 nm) [8]. It is interesting to note that many different new techniques have been recently developed to study EVs and their phenotypes. Among them, a detection platform combining a microfluidic device and surface-enhanced Raman spectroscopy (SERS), as well as ImageStream, dynamic light scattering (DLS), nanoparticle tracking analysis (NTA), cryo-transmission electron microscopy, label-free quantitative phase imaging, and atomic force microscopy, are all techniques recently used to study EVs [9,10,11,12,13,14,15,16,17]. Flow cytometry, because of its multiparameter properties and the capability to analyze thousands of events in a few seconds, theoretically has great potential for the study of EVs. A recently published flow cytometry method allows analyzing untouched body fluids by polychromatic flow cytometry, largely ameliorating the sensitivity of EV detection [18,19].

EVs have been implicated in different pathological processes, such as endothelial damage, atherogenesis, thrombosis [20], mucosal immunity dysregulation and inflammatory response [21], diabetes [22], bacteria–host interactions [23], neurological and proteinopathies-driven neurodegenerative disorders, autoimmune diseases, as well as fetal–maternal communication [24,25,26]. Recent studies have demonstrated an increased number of circulating EVs in a variety of conditions characterized by multi-organ impairment and/or damage, such as obesity, type 1 and type 2 diabetes, and metabolic syndrome, and they have been linked to macro- and microvascular dysfunction [22]. Increased numbers of circulating EVs have been implicated in tumor growth and metastasis in some types of cancer [27,28]. They are primarily thought to carry growth factors promoting angiogenesis, and to transfer receptors, such as CD41, to circulating tumor cells directly, promoting adhesion [29].

It is already known, in fact, that circulating EVs, which are released theoretically by all types of normal and diseased tissues, dynamically reflecting the peripheral organ and tissue status, have been proposed as reliable biomarkers of disease processes, even for liquid biopsy purposes and as tools to deliver new therapies [3]. It has been demonstrated that in pancreatic cancer, the levels of the DNA carried by EVs better predict outcomes than circulating DNA [30]. The DNA fragments conveyed by circulating EVs have been proposed as good representatives of the tumor genome in paraganglioma or pheochromocytoma patients [31]. Children with autism spectrum disorders display higher levels of mitochondrial DNA than controls [32]. Furthermore, platelet-derived EVs have been shown to exhibit anti-apoptotic effects and may also help the circulating neoplastic cells to evade immune surveillance in a similar manner to platelets [33]. It has been also reported that EVs play a key role in inflammatory disease models, such as inflammatory bowel disease (IBD) [34].

Celiac disease (CD) is a gluten-triggered immune-mediated disorder, characterized by the inflammatory insult of the enteric mucosa following local lymphocytic infiltration, eventually resulting in villous atrophy. This process, principally affecting genetically predisposed individuals, has been linked to extra-intestinal manifestations, both autoimmune (e.g., autoimmune thyroiditis) and immune-mediated (e.g., gluten ataxia or epilepsy) [35,36]. The mechanisms linking the mucosal insult to the systemic manifestations of celiac disease are not completely understood and may involve common genetics and triggers, as well as disease-specific signaling. Coupled with the fact that the extra-intestinal manifestations of celiac disease are not always linked to disease severity, malabsorption, or gluten-free diet efficacy in controlling mucosal damage, it can be hypothesized that hitherto unrecognized EV-mediated crosstalk mechanisms may potentially play a role in the pathogenesis and evolution of celiac disease. To the best of our knowledge, only one paper studied the involvement of EVs in CD, demonstrating that EVs carry miRNAs (i.e., miR-99b-3p, miR-197-3p, miR-374b-5p) involved in the hypersensitivity to gluten, possibly contributing to CD-associated diseases [37]. In this study, we aimed to assess and characterize circulating EVs by flow cytometry in newly diagnosed celiac disease patients, as well as in patients already on an adequate gluten-free regimen (GFD), applying a novel approach based on polychromatic flow cytometry [18,19] that allows the identification and enumeration of different EV sub-types.

## 2. Materials and Methods

### 2.1. Patients

This protocol was approved by the local Ethics Committee (document approved in 2017, session number 02, 6 April 2017, code CCMCDP1; http://comitatoetico.unich.it/, accessed on 1 October 2022), and included subjects that signed an informed consent before enrollment. In this study, we enrolled consecutive adult anti-tissue transglutaminase (anti-tTG)-positive biopsy-proven celiac disease patients at initial diagnosis to the Regional Center for Adult Celiac Disease at the “SS. Annunziata” University Hospital of Chieti, Italy. According to current guidelines [35], all patients underwent esophagogastroduodenoscopy (EGDS). Five duodenal biopsies, including one in the bulb, were obtained for histological examination, oriented on cellulose paper to avoid artifacts. Histology was considered positive for lesions of grade ≥ B1, according to the Corazza–Villanacci classification [38]. All included subjects with celiac disease were confirmed to display HLA-DQ2/8 positivity. In the same period, we included age- and sex-matched celiac disease patients undergoing a gluten-free diet for at least 12 months before inclusion. Compliance with the dietary regimen was confirmed by demonstrating negative serum anti-transglutaminase titers, as per current guidelines. Furthermore, we included age- and sex-matched healthy controls (HC), selected among those undergoing EGDS for reasons unrelated to celiac disease (e.g., heartburn, dyspepsia) with endoscopically normal stomach and small intestine. Exclusion criteria were: IBD or concomitant inflammatory disorders, pre-neoplastic conditions/cancer, vascular disease, diabetes, major recent surgery, use of steroids, NSAIDs, and statins.

### 2.2. Flow Cytometry Analysis

Circulating EVs were identified, analyzed, and enumerated in untouched whole blood samples, as described in a recently patented method that has been validated elsewhere by using specific required approaches [18,19,27,39,40]. The main advantage of the here-proposed protocol relies on the fact that it does not require any pre-analytical enrichment step and, therefore, it is a rapid tool for the identification of 0.1–1 µm EVs in untouched body fluids, also allowing the obtainment of reliable EV counts [18,19]. In detail, peripheral blood (PB) was drawn by venipuncture (21 Gauge needle) in sodium citrate tubes and processed within 4 h from collection. For each analysis, 0.5 µL of lipophilic cationic dye (LCD; Becton Dickinson-BD Biosciences, San Jose, CA, USA; Catalog, #626267, Custom Kit), a pan-EV dye recently developed in our laboratories and used elsewhere [18,19,27,39,40,41], was added to 195 µL of 1× Annexin V binding buffer (Becton Dickinson-BD Biosciences, San Jose, CA, USA), together with a mix of reagents, as detailed in Table 1. Then, 5 µL of PB was added to the reagent mix and incubated at RT for 45 min in the dark, and 500 µL of 1× Annexin V binding buffer (BD Biosciences) was finally added to each tube. We acquired 1 × 10^6^ events/sample using flow cytometry (FACSVerse, Becton Dickinson-BD Biosciences, San Jose, CA, USA, three lasers, eight-color configuration) by setting a threshold on the channel in which the lipophilic cationic dye emits (APC channel). Amplifier settings for forward scatter (FSC) and side scatter (SSC), as well as for any fluorescence channel, were set in logarithmic mode, and all parameters were visualized as height (H) signal. “PLT-free area” on scatter parameters was validated to be dimensionally corrected (~0.1–1 µm) by Rosetta Calibration beads (Exometry, Amsterdam, The Netherlands) as previously published [42].

Instrument performances and data reproducibility were implemented and checked by the Cytometer Setup and Tracking Module (Becton Dickinson-BD Biosciences, San Jose, CA, USA). In order to evaluate non-specific fluorescence, Fluorescence Minus One (FMO) controls were used. Compensation was assessed using CompBeads (Becton Dickinson-BD Biosciences, San Jose, CA, USA ) and single-stained samples. Data were analyzed using FACSuite v1.06.5230 (Becton Dickinson-BD Biosciences, San Jose, CA, USA) and FlowJo v10.7.2 (Becton Dickinson-BD Biosciences, San Jose, CA, USA) software.

### 2.3. Gating Strategy for EV Detection and Sub-Typing by Polychromatic Flow Cytometry

**Figure 1:**
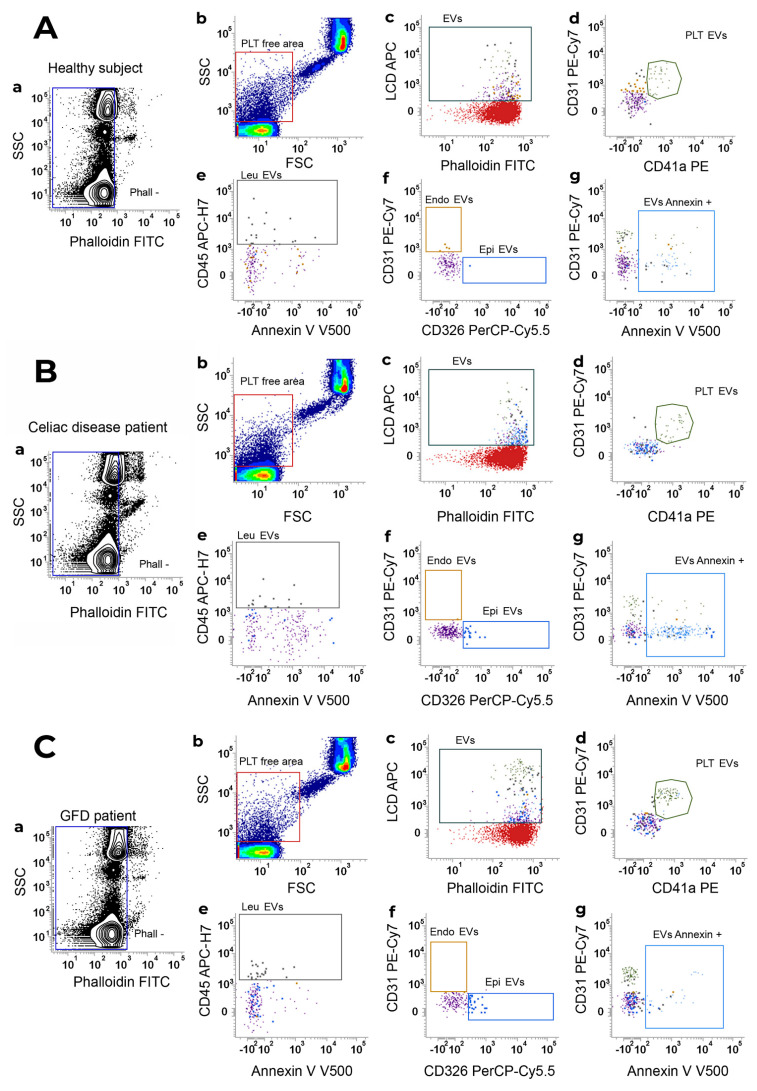
Gating strategy for EV identification. EV identification and sub-typing in (**A**) healthy subjects, (**B**) Celiac disease patients and (**C**) celiac disease patients under a gluten-free diet (GFD patient). (**a**) Phalloidin-negative events were firstly gated and (**b**) analyzed for their SSC-H/FSC-H parameters. The scatter area identified on the basis of MegaMix Plus beads (broadly 0.1–1 µm) was selected and named platelet (PLT)-free area. (**c**) Events were then analyzed on a Phalloidin-H/LCD-H dot-plot and phalloidin– LCD+ vesicles were gated and identified as EVs. EVs were finally sub-typed: (**d**) platelet-derived EVs (PLT-EVs) were gated on the basis of their combined positivity to CD31 and CD41a. The gate “NOT-PLT-EVs” was obtained and, in such a population, (**e**) Leukocyte-derived EVs (Leu-EVs) were identified as CD45+ events. By creating a logical gate “Not-Leu-EVs”, (**f**) endothelial-derived EVs (CD31+, Endo-EVs) and epithelial-EVs (CD326/EpCAM+, Epi-EVs) were also gated. (**g**) Annexin V positivity was also assessed on an Annexin V-H/CD31-H dot-plot, and the gate was then applied to any identified EV subset. Data are representative of all peripheral blood samples analyzed by flow cytometry.

The gating strategy for EV identification in healthy subjects (Figure 1A), celiac disease (Figure 1B), and GFD patients (Figure 1C) is shown in Figure 1. For each analyzed sample, events were characterized for their Phalloidin signal, and Phalloidin-negative particles (intact events; Figure 1(Aa,Ba,Ca) were analyzed for FSC-H and SSC-H parameters (Figure 1Ab,Bb,Cb). In detail, by running MegaMix Plus SSC and FSC beads (BioCytex, Marseille, France), the area containing all events with sizes broadly in the range of 0.1–1 µm was identified on a dot-plot FSC-height (FSC-H) versus SSC-height (SSC-H). This region was named platelet (PLT)-free area (Figure 1Ab,Bb,Cb). The “PLT-free area” was then tuned on scatter parameters and it was dimensionally corrected by Rosetta Calibration beads (Exometry, Amsterdam, The Netherlands) as previously published [42]. All the Phalloidin-negative events (Phall−), falling in the “PLT-free area”, previously established by the beads were selected (Figure 1Ab,Bb,Cb) and analyzed for their positivity to the lipophilic cationic dye (LCD; which identifies the whole EV compartment, Figure 1Ac,Bc,Cc). Phalloidin– LCD+ were then sub-typed as: platelet (PLT)-derived EVs (CD41a+/CD31+, Figure 1Ad,Bd,Cd); leukocyte (Leu)-derived EVs (CD45+, in the “NOT PLT-EVs” gate, Figure 1Ae,Be,Ce); endothelial (Endo)-derived EVs (CD31+, in the “NOT Leu-EVs” gate, Figure 1Af, Bf and Cf), and epithelial (Epi)-derived EVs (CD326/EpCAM+, in the “NOT Leu-EVs” gate, Figure 1Af,Bf,Cf). Finally, the whole EV compartment, as well as each EV sub-population, was analyzed for the surface presence of phosphatidylserine by monitoring the Annexin V binding (Figure 1Ag,Bg,Cg).

### 2.4. Statistical Analysis

Data were expressed as mean ± standard deviation (SD) or mean and 95% confidence interval (95%CI); statistical differences of continuous variables between groups, log-transformed where appropriate, were evaluated by means of Student’s *t*-test or ANOVA (Welch correction was used when applicable), with post hoc Bonferroni or Dunnett’s T3 test, and considered significant for *p* < 0.05. Ratios and categorical variables were evaluated by the chi-squared test. Linear regression analysis was used to assess continuous independent variables. Correlations between continuous independent variables were assessed by the Pearson correlation coefficient (*r*).

## 3. Results

### Patient Characteristics and EV Concentrations

We evaluated 22 adult celiac disease patients at diagnosis (mean age = 40 ± 14 years, sex ratio F/M = 8:1), 11 age- and sex-matched celiac disease patients under GFD, and 22 healthy controls. The mean anti-tTG levels at inclusion were 13.7 ± 10.8 times ULN in newly diagnosed celiac disease patients. The gluten-free regimen mean duration was 4.6 ± 1.8 years; adherence was assessed by anti-tTG negativity at the time of inclusion in this study. The histological and laboratory characteristics of patients are shown in Table 2. Total atrophy was expectedly more prevalent in newly diagnosed celiac disease patients (Grade B2 41% vs. 12.5%, *p* = 0.049).

**Figure 2:**
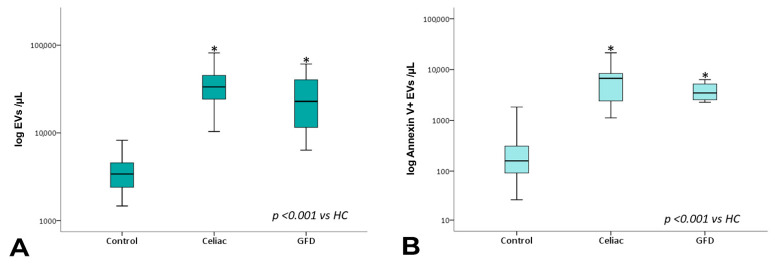
Total EV and annexin V+ EV counts. Box plots represent total (**A**) and total Annexin V+ (**B**) EV concentrations (EVs per µL, log-transformed) in peripheral blood obtained from controls, celiac, and celiac-GFD patients. The horizontal line represents the median value. The asterisk (*) identifies statistically significant differences.

EV counts are reported in Table 3. Overall, the mean number of total circulating EV counts was significantly higher in both newly diagnosed celiac disease patients and patients under GFD than in healthy controls. This was also the case for total annexin V+ EV counts (Table 3, Figure 2).

**Figure 3:**
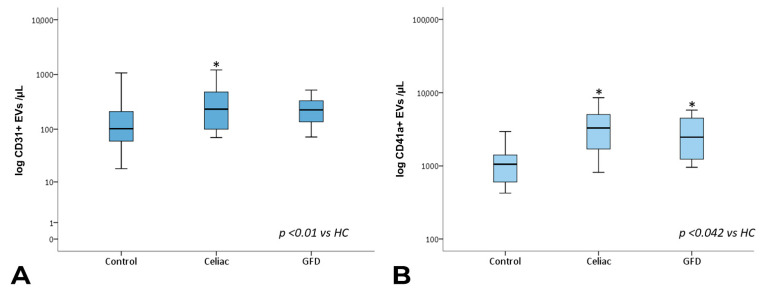
Total CD31+ and CD41a+ EV counts. Box plots represent CD31+ (**A**) and CD41a+ (**B**) EV concentrations (EVs per µL, log-transformed) in peripheral blood obtained from controls, celiac, and GFD patients. The horizontal line represents the median value. The asterisk (*) identifies statistically significant differences.

**Figure 4:**
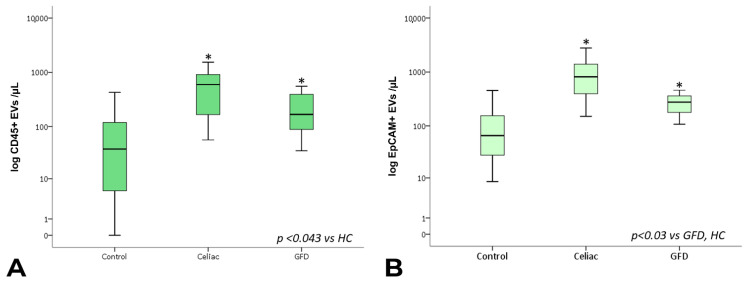
Total CD45+ and EpCAM+ EV counts. Box plots represent CD45+ (**A**) and EpCAM+ (**B**) EV concentrations (EVs per µL, log-transformed) in peripheral blood obtained from controls, celiac, and GFD patients. The horizontal line represents the median value. The asterisk (*) identifies statistically significant differences.

The analysis of the EV sub-populations showed that EpCAM+ EVs, which are EVs of epithelial origin, CD45+ leukocyte-derived EVs, and CD41a+ and CD41a+ Annexin V+ platelet-derived EVs, as well as CD31+ endothelium-derived EVs, were significantly increased in celiac disease patients compared with healthy subjects. Notably, CD45+ and CD41a+ EVs showed significantly higher counts in celiac disease patients regardless of gluten exposure (Figure 3 and Figure 4).

**Figure 5:**
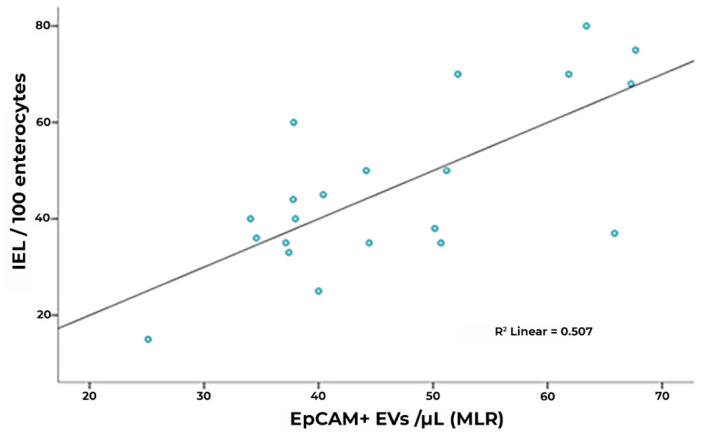
Correlation of IEL concentration and R model-predicted EpCAM+ counts (unstandardized predicted value) in CD. In a multiple linear regression model including total, EpCAM+, and CD31+, CD45+ and CD41a+ counts, EpCAM+ EVs represent a statistically significant predictor (IEL: intraepithelial leukocytes).

Interestingly, however, EpCAM+ EV concentrations were significantly lower in subjects under gluten exclusion, although they displayed higher counts than healthy subjects (Figure 4B). In the case of EVs of leukocyte and platelet origin, correction using total counts of circulating leukocytes/lymphocytes and platelets, respectively, did not change the observed differences in EV expression. In a multiple linear regression model including the total, EpCAM+, CD31+, CD45+, and CD41a+ EV counts, EpCAM+ EVs were statistically significant predictors of intraepithelial leukocytes (Figure 5, Supplementary Tables S1–S3). EpCAM+ EVs were more linked to superficial enterocyte monolayer infiltration and damage.

## 4. Discussion

To the best of our knowledge, this is the first study linking celiac disease with an increased number of circulating EVs. Our data show that newly diagnosed celiac disease patients show higher numbers of circulating EVs than age- and sex-matched healthy subjects, owing in part to the increase in the endothelial- (CD31+), platelet- (CD41a+), epithelial-(EpCAM+), and leukocyte-derived (CD45+) EVs. Moreover, our data suggest that the epithelial- and endothelial-derived EVs are responsive to gluten exclusion, potentially reflecting a decrease in tissue-specific signaling or cell apoptosis in treated subjects. However, whether this represents a marker of tissue damage or an up-regulation of homeostatic immune mechanisms is unclear, as increases in EV numbers have also been observed in protective immune activation and tissue repair in some cases [5].

While no data are available on celiac disease, studies on chronic inflammatory disorders of the bowel show that patients with active Crohn’s disease have increased counts of total circulating EVs compared with healthy controls, with increased counts of EVs of endothelial, platelet, and leukocyte origin [34,43,44]. Furthermore, disease activity, as measured by the Harvey–Bradshaw index, has been reported to correlate with counts of total EVs, as well as platelet- and endothelial-derived EVs in some studies [34]; however, others have not confirmed these findings [44]. Similarly, data on the effect of anti-TNFα treatment on circulating EVs are also conflicting [43,44]. Overall, existing literature points to a potential role of circulating EVs in inflammatory signaling and endothelial damage. Specifically, regarding the EpCAM+ EV sub-population, which stems from the epithelium, our data suggest an even more specific correlation to intestinal insult by showing not only an increase in EV numbers in celiac disease patients compared with healthy subjects but also a significant correlation to the degree of lymphocytic infiltration of the mucosa. Interestingly, EpCAM+ EV counts were significantly different between celiac disease patients undergoing gluten-exclusion diets and healthy controls, possibly suggesting that EpCAM+ EVs are markers of mucosal damage. However, the specific nature of the epithelial EV compartment increase remains to be further elucidated, as it cannot be excluded that it represents a defensive mechanism [45], which is not needed in patients undergoing GFD, rather than an expression of tissue damage, as found in other epithelia [46]. In this context, an experimental in vitro study suggested a potential role of gliadin in phospholipid giant vesicle generation and lipid bilayer properties [47]. Similarly, endothelial-derived CD31+ EVs were increased in celiac disease and were also linked to villous atrophy; however, they were not significantly higher in patients under a gluten-free diet or associated with IEL concentrations. In light of these data, it might be speculated that CD31+ EVs are in part linked to deep endothelial damage within the impaired mucosa, more readily present in higher grades of villous atrophy, while EpCAM+ EVs are more linked to superficial enterocyte monolayer infiltration and damage. Gut–vascular barrier impairment has already been demonstrated in celiac disease and has been shown to be independent of mucosal barrier dysfunction [48]. On the contrary, increased numbers of leukocyte- and platelet-derived EVs seem typical of celiac disease at any stage. These sub-populations persist after gluten exclusion, suggesting a potential implication in long-term disease-specific systemic signaling. Interestingly, a persistence of increased Annexin V+ EV counts, partially from the platelet-derived compartments, was also noted. Annexin V, a marker of phosphatidylserine membrane expression, has been linked to EV activation, particularly in thrombosis [44] but also of cellular apoptosis [5]. However, their roles as effectors of damage or mediators of protective immune response is unclear. For instance, endothelium-derived EVs have been shown both to correlate to endothelial dysfunction [49] and to promote endothelial tissue repair [50] in particular settings. Celiac disease represents a unique paradigm of a disease characterized by gluten-induced enteropathy, in which removal of the trigger is generally followed by remission of laboratory values and clinical manifestations, as well as a gradual regression of atrophy after gluten exclusion. As such, it could be a useful model of disease–trigger interaction in EV research. However, a number of studies [51,52,53] have shown that a return to the normal histology of the duodenal mucosa can be observed in a proportion of subjects varying from 20 to 50%. Celiac disease is also associated with both autoimmune- and immune-mediated complications, whose development is frequently independent of the activity, severity, or treatment response. Our data point toward a potential implication of EVs in systemic signaling, both at the initial and at the later stages of celiac disease. Further studies are needed to better understand the specific patterns, associations, and effects of EVs and their potential role in celiac disease-associated conditions of non-autoimmune origin. In recent years, novel approaches for the identification of novel biomarkers of intestinal mucosal disease and barrier impairment have been proposed. These range from novel histological markers, bacterial/microbiomic and metabolomic signatures, hormonal/growth-factor patterns, and stem/progenitor cell mobilization in the peripheral blood to the EV compartment and its functional content [54]. For example, micro-RNAs have been the focus of research as mediators of cellular crosstalk in many disease models, including inflammatory disease, celiac enteropathy, and gluten-related disorders. Some miRNAs show differential expression in the human small intestine, such as miR-449a, which is upregulated in children with celiac disease, both off and on GFD, and could be a distinctive feature of the disease [55]. The profiling of peripheral blood miRNAs has been shown to differentiate patients with gluten-related disorders from healthy controls [56]. miRNA expression is also dependent on the severity of mucosal damage and could modulate the expression of other mediators involved in innate and adaptive immunity [57]. However, miRNAs, among other mediators, can travel both freely in the peripheral blood and within EVs [3].

These concepts have been researched primarily in the context of inflammatory bowel disease, in part because of a more pressing need for disease-specific markers of inflammation and progression. However, these paradigms might be useful for the diagnosis and monitoring of other immune-mediated intestinal diseases, including celiac disease. In celiac disease, mucosal recovery generally follows anti-tTG titer normalization after GFD introduction; however, it has been shown that mucosal damage can persist long after serological remission and, in some cases, tTG-negativity can be present along non-responsive or frankly refractory disease [35]. Furthermore, intestinal diseases have frequent extra-intestinal complications and many broadly immune-mediated but ultimately unknown triggers and mechanisms. For these reasons, the identification of a biomarker, such as EVs, that is able to reveal the general status of the organism in real time, may be useful in the correct management of CD patients, shedding light on the possible etiology of refractory disease.

## 5. Conclusions

Here, we demonstrated that the applied flow cytometry protocol allows a rapid and real-time detection of circulating EVs, representing a highly promising tool for further clinical applications. Furthermore, these data show that EVs represent novel and potentially powerful celiac disease-specific biomarkers, reflecting the real-time pathophysiological status of any cellular sub-type.

## Figures and Tables

**Table 1:** List of flow cytometry specificities and reagents ^1^.

Detection	Fluorochrome	Ab Clone	Catalog	Amount per Test
Phalloidin	FITC		626267 (custom kit)	0.5 µL
CD41a	PE	HIP8	626266 (custom kit)	5 µL
CD31	PE-Cy7	WM59		5 µL
CD45	APC-H7	2D1	560178	2 µL
EpCAM/ CD326	PerCP-Cy5.5	EBA-1	347199	5 µL
Annexin V	V500		561501	1 µL

^1^ Keys: Fluorescein-5-isothiocyanate (FITC), R-phycoerythrin (PE); PE-Cyanine 7 (Cy7), Allophycocyanin-Hilite^®^7 (APC-H7), Peridinin–Chlorophyll–protein–Cyanine 5.5 (PerCP-Cy5.5). Becton Dickinson-BD Biosciences, San Jose, CA, USA.

**Table 2:** Histological and laboratory findings ^2^.

Parameter	Healthy Controls (HD) *n* = 22	Celiac Disease (CD) *n* = 22		GFD *n* = 11	Significance
Histology grading		A	**9.0%**	37.5%	*p* < 0.049 vs. non-atrophy prevalence in GFD and severe atrophy prevalence in GFD
		B1	50.0%	50.0%	
		B2	**41.0%**	12.5%	
IEL × 100 enterocytes		**52 ± 18**		35 ± 6	*p* = 0.004 vs. GFD
Anti-t-TG (×ULN)	0.6 ± 0.8	**13.7 ± 10.8**		0.7 ± 0.7	*p* = 0.01 vs. GFD, HC
Anti-deamidated gliadin IgA (×ULN)	0.2 ± 0.2	**2.5 ± 4.5**		0.3 ± 0.3	*p* = 0.039 vs. GFD, HC
Anti-deamidated gliadin IgG (×ULN)	1 ± 0.1	8.3 ± 9.1		0.6 ± 0.8	*p* = n.s.
Hemoglobin (×ULN)	**1.2 ± 0.06**	1.03 ± 0.09		1.08 ± 0.08	*p* = 0.026 vs. CD, GFD
MCV (fl)	**88.7 ± 2.6**	82.5 ± 9.6		88.4 ± 5.5	*p* = 0.037 vs. CD, GFD
Anemia	**4.5%**	**33.3%**		11.3%	*p* < 0.04
Serum ferritin (ng/mL)	**36.3 ± 45.2**	21.7 ± 26.1		28.8 ± 23.5	*p* = 0.031 vs. CD, GFD
Low serum ferritin	**4.5%**	**77.8%**		25.0%	*p* < 0.04 vs. prevalence of low serum ferritin in GFD and controls
ESR (mm/hr)	11.3 ± 5.6	10.6 ± 7.1		12.2 ± 4.1	*p* = n.s.
CRP (×ULN)	0.48 ± 0.19	0.56 ± 0.03		0.34 ± 0.28	*p* = n.s.
Fecal elastase (μg/g)	**499.1 ± 187.4**	180.6 ± 190.1		378.5 ± 108.2	*p* = 0.036 vs. CD, GFD
BMI (kg/m^2^)	**26.1 ± 5.1**	21.6 ± 3.5		22.5 ± 2.2	*p* = 0.043 vs. CD, GFD
Overweight	**54.5%**	11.1%		0%	*p* < 0.025

^2^ Group characteristics; absolute values are expressed as mean ± standard deviation and statistically significant values are indicated in bold (histology grading according to the Corazza–Villanacci classification, IEL: intraepithelial lymphocytes, t-TG: tissue transglutaminase, ULN: upper limit of normal, MCV: mean corpuscular volume, ESR: erythrocyte sedimentation rate, CRP: C-reactive protein, BMI: body mass index, n.s. = not significant).

**Table 3:** Differences in total and specific EV counts between groups ^3^.

EV Type	Healthy Controls (HC) *n* = 22	95% Confidence Interval		Celiac Disease (CD) *n* = 22	95% Confidence Interval		GFD *n* = 11	95% Confidence Interval		Significance
	Mean	Lower Bound	Upper Bound	Mean	Lower Bound	Upper Bound	Mean	Lower Bound	Upper Bound	
**Total**	3696	2895	4717	**35944**	23735	54432	**25439**	13766	47012	*p* < 0.001 vs. HC
**Total** **annexin V+**	153	66	357	**5485**	3293	9138	**4878**	2173	10950	*p* < 0.001 vs. HC
**CD31+ (endothelium)**	83	39	176	**293**	179	481	209	108	403	*p* < 0.01 vs. HC
**CD31+** **annexin V+**	27	16	80	91	53	158	36	16	85	n.s.
**CD41a+ (platelet)**	964	624	1489	**3729**	2281	6096	**2166**	1132	4147	*p* < 0.042 vs. HC
**CD41a+** **annexin V+**	86	44	155	**1589**	831	3041	**933**	361	2412	*p* < 0.009 vs. HC
**CD45+ (leukocyte)**	56	27	116	**394**	211	737	**164**	116	338	*p* < 0.043 vs. HC
**CD45+** **annexin V+**	26	10	72	95	50	182	41	11	155	n.s.
**EpCAM (CD326)+ (mucosa)**	87	44	173	**770**	453	1307	**257**	158	417	*p* < 0.03 vs. GFD, HC
**EpCAM (CD326)+** **annexin V+**	12	5	42	61	27	136	33	14	80	n.s.

^3^ Shown values are back-transformed from log values. Significant differences in bold; CI: confidence interval, n.s. = not significant.

## Data Availability

All datasets generated and analyzed are available from the corresponding author upon reasonable request.

## Supplementary Materials

The following supporting information can be downloaded at: https://www.mdpi.com/article/10.3390/nu15010071/s1, Table S1: Model Summary; Table S2: ANOVA; Table S3: Coefficients.
