# Supplementary material for: Circulating Extracellular Vesicles Are Increased in Newly Diagnosed Celiac Disease Patients
Source: Nutrients. 2022 Dec 23;15(1):71. doi: 10.3390/nu15010071 (PMC9824360; doi:10.3390/nu15010071)
Supplement: Supplementary file 1 [file nutrients-15-00071-s001.zip › nutrients-2070834-supplementary.pdf]

nutrients MDPI

Supplementary Table S1. Model Summary [b].

Model	R	R Square	Adjusted R Square	Std. Error of the Estimate
	0.712 [a]	0.507	0.343	14.162

[a] Predictors: (Constant), logEVtot, logEpCAM, logPLT, logCD31, logCD45; [b] Dependent Variable: IEL/100.

Supplementary Table S2. ANOVA [a].

Model	Sum of Squares	df	Mean Square	F	Sig.
Regression	3097.924	5	619.585	3.089	0.041 [b]
Residual	3008.362	15	200.557		
Total	6106.286	20			

[a] Dependent Variable: IEL/100; [b] Predictors: (Constant), logEVtot, logEpCAM, logPLT, logCD31, logCD45.

Supplementary Table S3. Coefficients [a].

Model	Unstandardized Coefficients		Standardized Coefficients	*t*	Sig.
	B	Std. Error	Beta		
(Constant)	−41.794	42.494		−0.984	0.341
logEVtot	−0.801	9.201	−0.018	−0.087	0.932
logCD31	−7.585	10.347	−0.160	−0.733	0.475
logCD45	−5.583	8.037	−0.197	−0.695	0.498
logPLT	12.513	9.322	0.290	1.342	0.199
logEpCAM	29.762	10.317	0.797	2.885	0.011

Supplementary Table S3 (continuation). Coefficients [a].

Model	95.0% Confidence Interval for B	
	Lower Bound	Upper Bound
(Constant)	−132.368	48.781
logEVtot	−20.412	18.811
logCD31	−29.640	14.469
logCD45	−22.713	11.548
logPLT	−7.357	32.382
logEpCAM	7.772	51.751

[a] Dependent Variable: IEL/100.
